# Zoonotic diseases transmitted from the camels

**DOI:** 10.3389/fvets.2023.1244833

**Published:** 2023-10-19

**Authors:** Abdelmalik Ibrahim Khalafalla

**Affiliations:** Development and Innovation Sector, Biosecurity Affairs Division, Abu Dhabi Agriculture and Food Safety Authority (ADAFSA), Abu Dhabi, United Arab Emirates

**Keywords:** camels, zoonotic diseases, camel-to-human transmission events, literature review, risks to human health

## Abstract

**Background:**

Zoonotic diseases, infections transmitted naturally from animals to humans, pose a significant public health challenge worldwide. After MERS-CoV was discovered, interest in camels was raised as potential intermediate hosts for zoonotic viruses. Most published review studies pay little attention to case reports or zoonotic epidemics where there is epidemiological proof of transmission from camels to humans. Accordingly, any pathogen found in camels known to cause zoonotic disease in other animals or humans is reported.

**Methods:**

Here, zoonotic diseases linked to camels are reviewed in the literature, focusing on those with epidemiological or molecular evidence of spreading from camels to humans. This review examines the risks posed by camel diseases to human health, emphasizing the need for knowledge and awareness in mitigating these risks.

**Results:**

A search of the literature revealed that eight (36.4%) of the 22 investigations that offered convincing evidence of camel-to-human transmission involved MERS, five (22.7%) Brucellosis, four (18.2%) plague caused by *Yersinia pestis*, three (13.6%) camelpox, one (4.5%) hepatitis E, and one (4.5%) anthrax. The reporting of these zoonotic diseases has been steadily increasing, with the most recent period, from 2010 to the present, accounting for 59% of the reports. Additionally, camels have been associated with several other zoonotic diseases, including toxoplasmosis, Rift Valley fever, TB, Crimean-Congo hemorrhagic fever, and Q fever, despite having no evidence of a transmission event. Transmission of human zoonotic diseases primarily occurs through camel milk, meat, and direct or indirect contact with camels. The above-mentioned diseases were discussed to determine risks to human health.

**Conclusion:**

MERS, Brucellosis, plague caused by *Y. pestis*, camelpox, hepatitis E, and anthrax are the main zoonotic diseases associated with human disease events or outbreaks. Transmission to humans primarily occurs through camel milk, meat, and direct contact with camels. There is a need for comprehensive surveillance, preventive measures, and public health interventions based on a one-health approach to mitigate the risks of zoonotic infections linked to camels.

## 1. Introduction

Zoonosis refers to the transmission of diseases or infections from vertebrate animals to humans in a natural manner (1). These incidences pose a significant challenge to public health on a global scale, primarily due to our intimate connection with animals in various contexts such as agriculture, companionship, and the natural world. Multiple sources have claimed that at least 60% of emerging infectious diseases (EIDs) affecting humans are naturally zoonotic, originating from animal hosts other than humans. Moreover, zoonotic pathogens have a twofold higher likelihood of being connected to the emergence of diseases than non-zoonotic pathogens (2–4). Climate change, land use change, agricultural practices, animal movements in search of food and water, international trade in livestock and their products, international travel and tourism, and pathogen evolution are all factors that have greatly influenced the emergence of zoonotic pathogens (5).

There are mainly two species of old-world camels: *Camelus dromedarius* (dromedary, Arabian, or one-humped camel), found in the Middle East, North, and Northern East Africa, and other parts of the world, and *Camelus bactrianus* (Bactrian, or two-humped camel), which is primarily found in northwestern China and southwestern Mongolia. Dromedaries have a long-standing association with human societies, particularly in the Middle East, North, and Northeast Africa. Their unique physiological adaptations, such as efficient water economy mechanisms and heat tolerance, enable them to survive in arid environments (6).

Over the past decade, the global camel population has experienced significant growth, with a particular increase in dromedaries (7, 8). This expansion, combined with socio-cultural practices, intensification of production, and concentration of camel farming, has increased the risk of zoonotic diseases associated with camels.

Throughout the dromedary camel belt from Mauritania to India, socio-cultural practices combined with weak public and animal health infrastructures, the recent intensification of the production, the concentration of the production around cities, and the change to camel farming favor the occurrence and spread of zoonosis. In recent years, camel production has been steady growth/expanding both in terms of intensification, such as the appearance of large dairy farms mainly in the Arabian Gulf, which represent a sizable growing business, or geographically as in Africa. For instance, the Borana communities of Kenya, traditionally known as cattle herders, have switched their preference from raising cattle to camels, as camels represent a viable option for these drought-affected ecosystems (7, 9). Furthermore, according to Gossner et al. (10), camel production has intensified in the Arabian Peninsula, making it easier for zoonotic diseases to “spill over” from camels to people. This would explain how the Middle East respiratory syndrome coronavirus (MERS-CoV) first appeared in the human population of the Arabian Peninsula.

The emergence of MERS-CoV infection in 2012 has attracted interest in camel diseases that can be transmitted to humans, and accordingly, several pathogens have been reported in the literature utilizing recent advances in molecular diagnostic techniques. The conclusion is that there is a risk to the public’s health since camels can contract many zoonotic infections. This ability of camels to be a potential source of diseases is a significant concern because more people consume their meat and milk. Therefore, knowledge of camel-associated zoonotic diseases and determining camels’ risks to human health are essential.

## 2. Current knowledge of zoonotic diseases associated with camels

Recent publications have focused on identifying camel pathogens without emphasizing diseases conclusively transmitted from camels to humans. Sazmand et al. (11) reviewed zoonotic parasites in dromedary camels. They identified 13 zoonotic parasites in camels, including *Trypanosoma species* (spp.), *Giardia duodenalis*, *Enterocytozoon* spp., *Balantidium coli*, *Toxoplasma gondii*, *Cryptosporidium* spp., *Blastocystis* spp., *Fasciola* spp., *Schistosoma* spp., *Echinococcus granulosus*, *Trichinella* spp., *Sarcoptes scabiei* var. *cameli*, and *Linguatula serrata*. However, to my knowledge, none of these parasitic pathogens have been reported as causing human diseases through camel transmission.

Similarly, Mohammadpour et al. (12) reviewed the zoonotic implications of camel diseases in Iran. They identified 19 important zoonotic diseases reported in Iranian camels, including 11 bacterial, four viral, and four parasitic diseases. However, the authors only considered the incidence of camel diseases caused by pathogens known to be zoonotic without addressing the actual transmission of these diseases from camels to humans.

In their review of camel-associated zoonoses in Kenya, Hughes and Anderson (13) mentioned zoonotic infections found in camels, similar to the studies discussed above. Among the 16 pathogens identified were *Trypanosoma* spp.*, E. granulosus*, *Brucella* spp., MERS-CoV, Rift Valley Fever virus, *Coxiella burnetii*, CCHF, *Dermatophilus congolensis*, and contagious ecthyma virus. According to Zhu et al. (14), Brucellosis, the Middle East Respiratory Syndrome Coronavirus (MERS-CoV), *E. granulosus*, and Rift Valley Fever (RVF) were the subjects of the majority of papers on zoonotic camel diseases (65%).

Therefore, compiling the most recent scientific data on camels’ direct contribution to the spread of zoonotic illnesses is necessary.

## 3. Searching for zoonotic diseases that camels have been shown to transmit to humans

Relevant publications on zoonoses associated with camels were searched using several search terms, including “zoonoses camels, diseases transmitted by camels, as well as specific disease terms such as MERS, *Yersinia pestis* in camels, rabies in camels, camelpox in humans/people, human brucellosis from camels.” The search was confined to authentic resources from repositories of popular databases: PubMed, Google Scholar, and SCOPUS.

This document only included compelling proof of camel-to-human transmission. All non-verified sources of information and studies describing the serological detection of human pathogens in camels were excluded from this review. Human zoonotic incidents in the dataset that have been identified as possibly involving camels are included in this study if (1) there is proven epidemiological data that the patient gets the infection from camels or their products or (2) sequence analysis of the causative agent from the patient and the camel samples revealed a close homology. For instance, this review does not include rabies, though it is the most important zoonotic disease worldwide. The reason is that no disease event confirmed the association of camels with human rabies.

After investigating rabies in camels in the Qassim region of central Saudi Arabia, Al-Dubaib (15) also came to the conclusion that camels do not transmit rabies to humans.

When evaluating the outcomes of infectious diseases associated with farm animals, one will find that diseases like MERS-CoV infection with a case fatality rate in humans of roughly 34% and that plaque caused by *Y. pestis* are predominantly disseminated from camels rather than cattle or small ruminants.

## 4. Major zoonotic diseases directly associated with the camels

**Figure 1:**
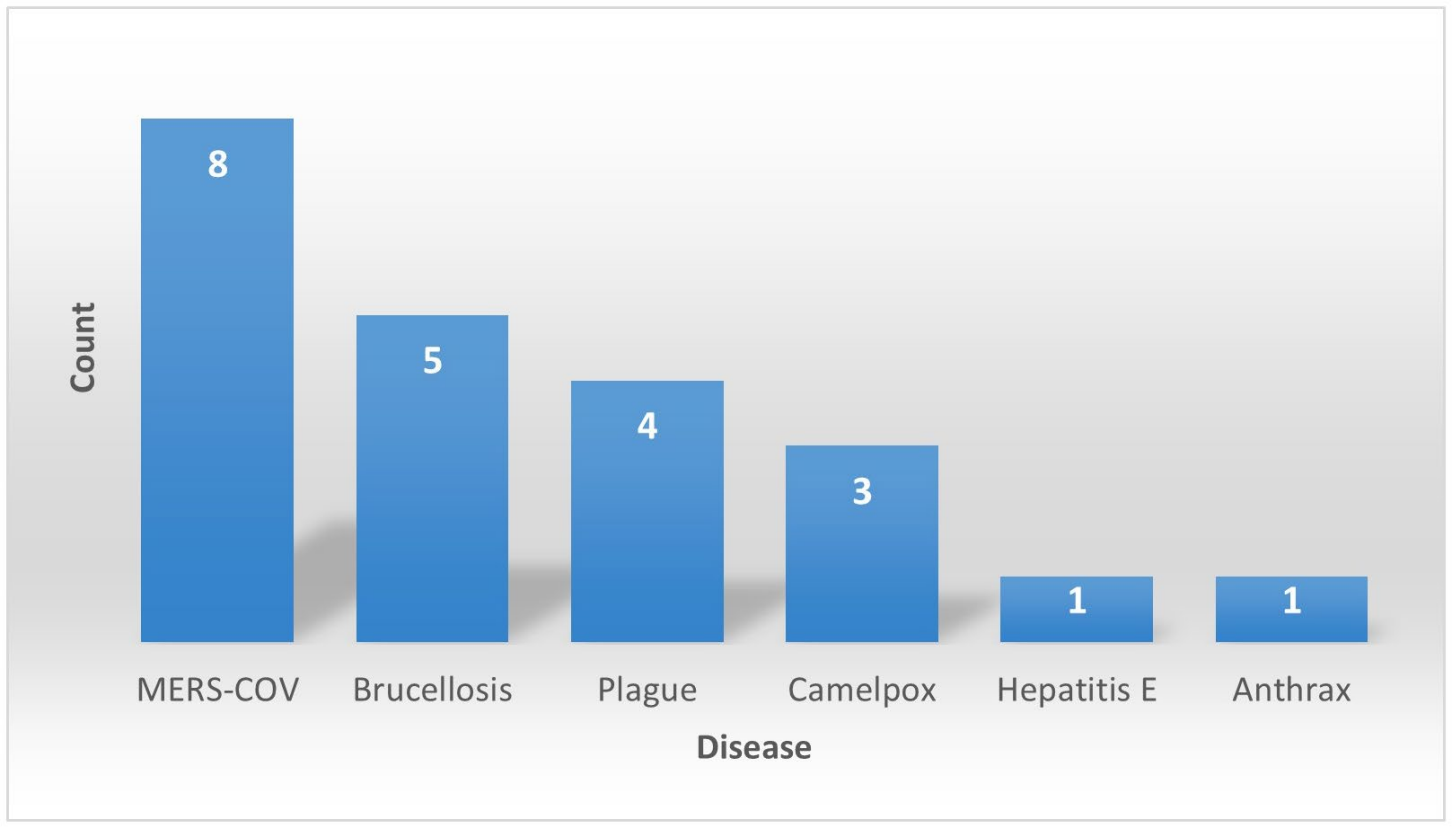
Camel diseases and records of human zoonotic occurrences.

Using the critical search terms, 872 scientific reports and articles were identified, 22 of which satisfied the inclusion criteria and were analyzed. Eight (36.4%) of the 22 publications examined dealt with MERS, five (22.7%) with Brucellosis, four (18.2%) with plague caused by *Y. pestis*, three (13.6%) with camelpox, one (4.5%) with hepatitis E, and one (4.5%) with anthrax (Table 1 and Figure 1).

**Table 1:** Details of camel-associated zoonotic diseases.

Disease	Country	Date	The zoonotic transmission proved by	Reference
MERS-CoV	Qatar	October 2013	Sequence analysis	Haagmans et al. (16)
MERS-CoV	Saudi Arabia	November 2013	Epidemiologic link and sequence analysis	Azhar et al. (17, 18)
MERS-CoV	Saudi Arabia	November 2013	Sequence analysis	Memish et al. (19)
MERS-CoV	UAE	July 2013–May 2014	Epidemiologic link and sequence analysis	Paden et al. (20)
MERS-CoV	UAE	February–May 2014	Sequence analysis	Al Muhairi et al. (21)
MERS-CoV	Saudi Arabia	2014–2016	Sequence analysis	Kasem et al. (22)
MERS-CoV	UAE	May 2015	Epidemiologic link and sequence analysis	Al Hammadi et al. (23)
MERS-CoV	Kenya	April 2018–March 2020	Sequence analysis	Ngere et al. (24)
Brucellosis	UAE	2008	Epidemiologic link and sequence analysis	Schulze zur Wiesch et al. (25)
Brucellosis	Israel	June 2011	Epidemiologic link and lab analysis	Shimol et al. (26)
Brucellosis	Israel	July–November 2016	Sequence analysis	Bardenstein et al. (27)
Brucellosis	Somalia, Ethiopia, Djibouti	2007 and 2013	Epidemiologic link	Rhodes et al. (28)
Brucellosis	Qatar	February 2015	Epidemiologic link	Garcell et al. (29)
Plague (*Y. pestis* infection)	Libya	February 1976	Epidemiologic link	Christie et al. (30)
Plague (*Y. pestis* infection)	Saudi Arabia	February 1994	Epidemiologic link	Bin Saeed et al. (31)
Plague (*Y. pestis* infection)	Afghanistan	December 2007	Epidemiologic link	Leslie et al. (32)
Plague (*Y. pestis* infection)	Jordan	February 1997	Epidemiologic link	Arbaji et al. (33)
Camel pox	Somalia	1987	Epidemiologic link	Kriz (34)
Camel pox	India	April–May 2009	Epidemiologic link	Bera et al. (35)
Camel pox	Sudan	September–December 2014	Epidemiologic link	Khalafalla and Abdelazim (36)
Hepatitis E infection	UAE	July 2012	Epidemiologic link and sequence analysis	Lee et al. (37)
Anthrax	Sudan	February 1988	Epidemiologic link	Musa et al. (38)

**Figure 2:**
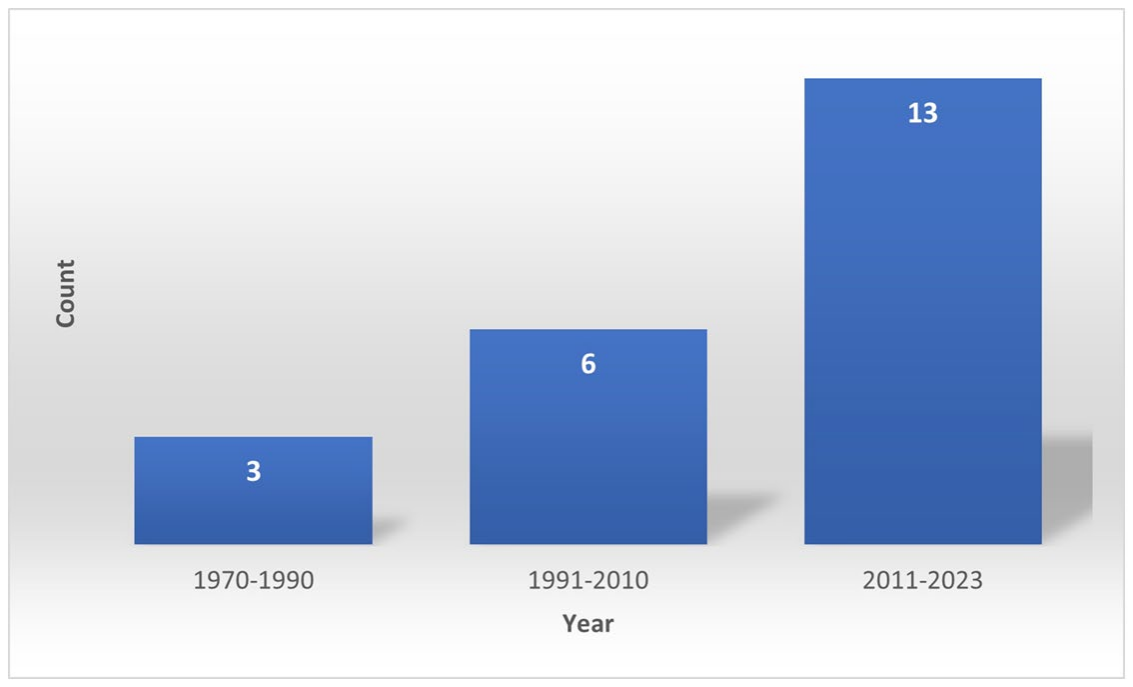
Distribution of records on camel zoonotic disease events by year of occurrence.

While some identified diseases, like MERS-CoV and HEV infections, do not manifest in the camel but can still seriously affect humans, others, like camelpox and brucellosis, infect both hosts. The first documented zoonotic disease associated with camels, a case of Plague (*Y. pestis* infection), was reported in Libya in February 1976 (30), while the most recent case was an outbreak of MERS-CoV in 2018 and 2020 (24). Figure 2 shows a constant increase in reporting these zoonotic diseases from 1980 to 2022, with the most recent period, from 2010 to the present, accounting for 59% of the reports.

**Figure 3:**
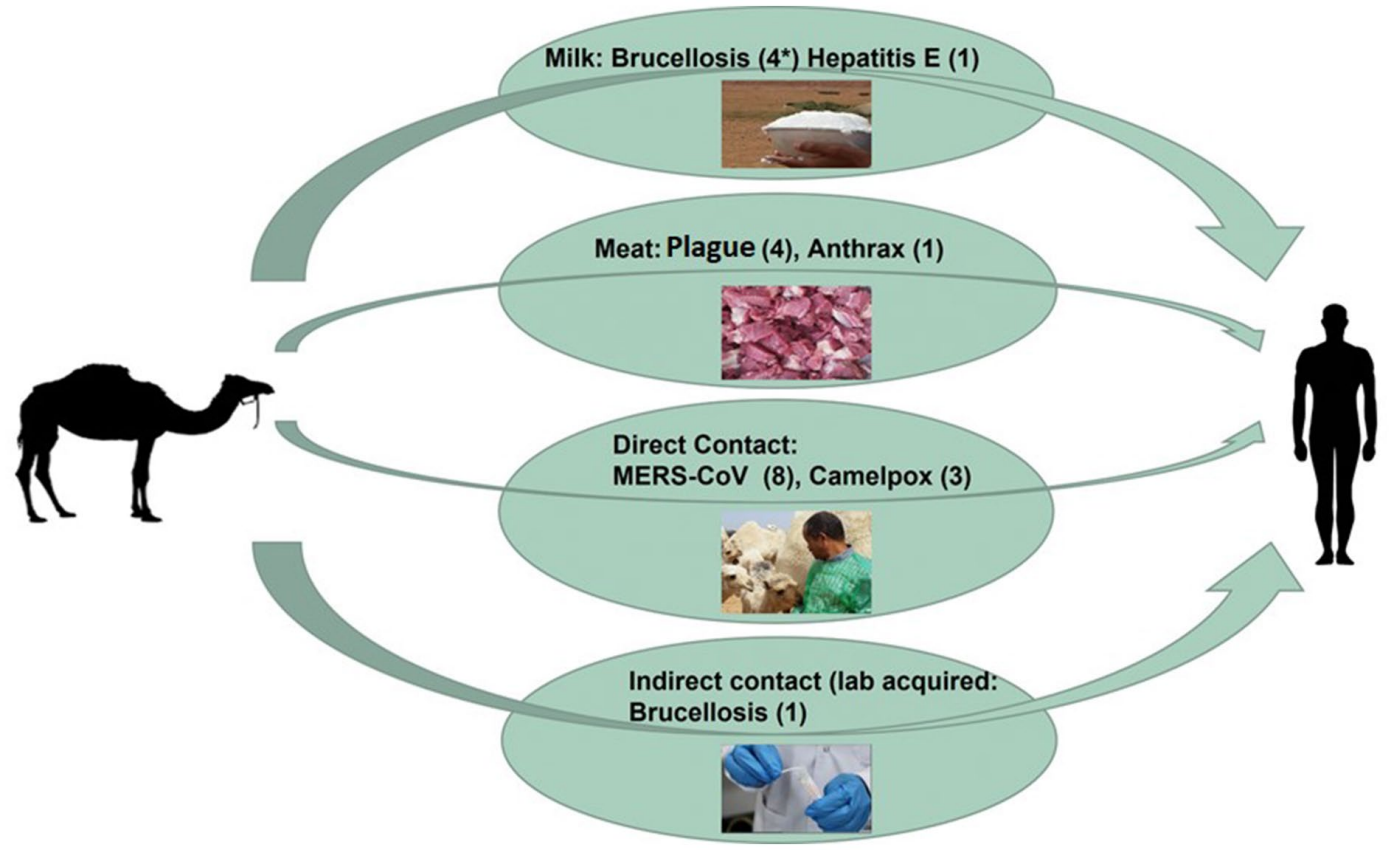
Overview diagram of camel-borne zoonotic disease transmission routes. The overall number of zoonotic disease incidents is displayed between two brackets (*).

**Figure 4:**
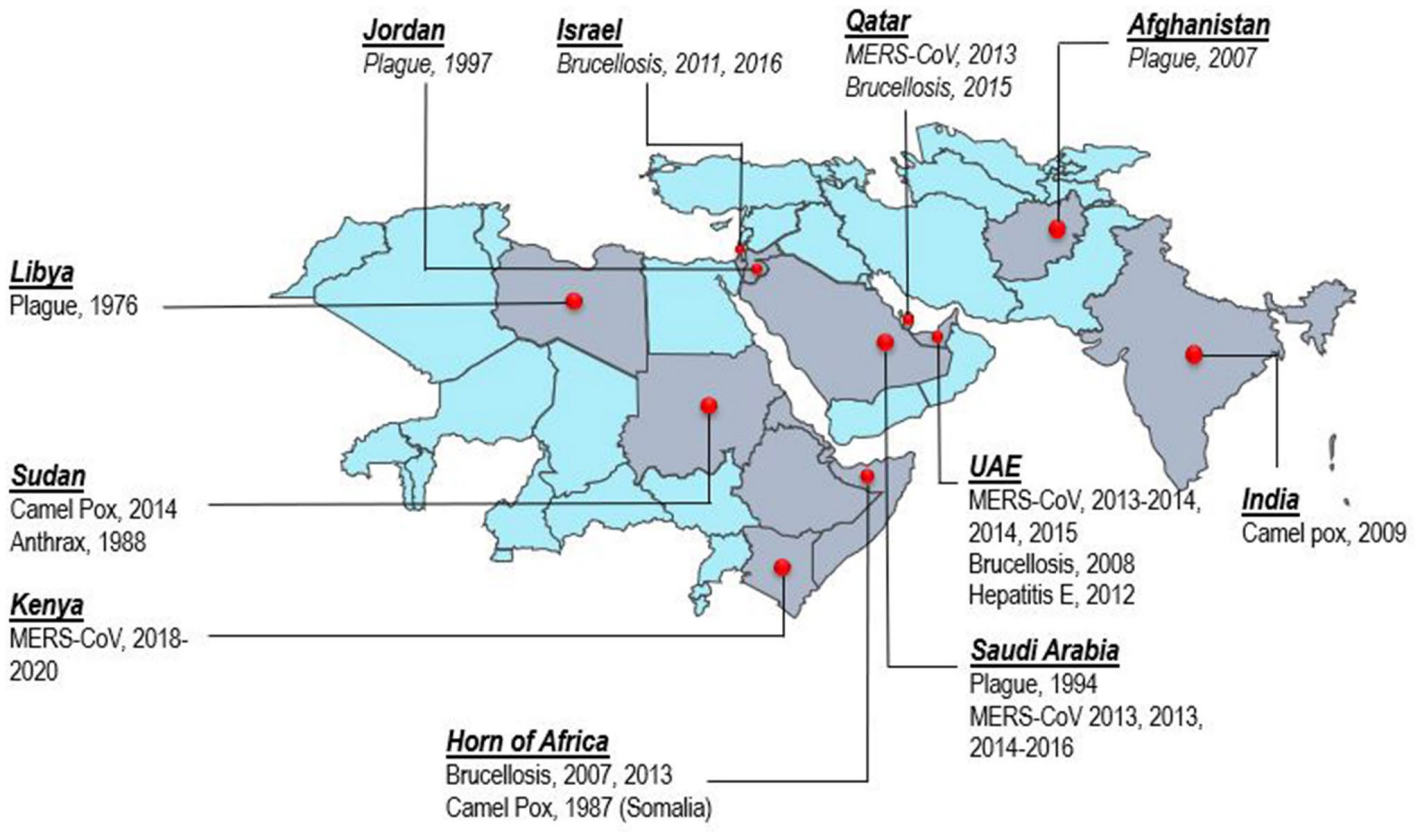
Map of camel belt displaying countries where zoonotic disease transmission from dromedary camels to humans has been documented. The map depicts zoonotic diseases per country with the year of reporting.

Consumption of camel milk, meat, or direct or indirect contact with camels is an essential source of human zoonotic disease transmission (Figure 3). The countries where the transmission of zoonotic diseases from dromedary camels to humans has been confirmed are shown in Figure 4. Zoonotic diseases are displayed on the map by country, and the year they first emerged. These countries are located in the camel belt and include seven Asian and six African countries, extending from Libya in the west to India in the east.

### 4.1. MERS-CoV infection

Middle East Respiratory Syndrome (MERS) is a viral respiratory system disease caused by the MERS coronavirus (MERS-CoV), which first emerged in Saudi Arabia in 2012. Structured coalescent models demonstrated that long-term MERS-CoV evolution only occurs in camels. The seasonally variable zoonotic transmission of viruses from camels is the primary cause of human epidemics in the Arabian Peninsula (39). Dromedary camels appear to be the only animal host responsible for the spillover of human infections, although bats and alpacas can act as potential reservoirs for MERS-CoV (40). Phylogenetic and sequencing data strongly suggest that MERS-CoV likely originated from bat ancestors due to a recombination event in the spike protein, which may have occurred in dromedary camels in Africa before being transported to the Arabian Peninsula via camel trade routes (41). Two of the initial MERS-CoV cases in Qatar were men who attended Doha’s main livestock market and the neighboring central slaughterhouse. According to Farag et al. (42), camel slaughterhouses are high-risk areas for human exposure where MERS-CoV is circulating. The research community responded quickly to the high fatality rates from MERS outbreaks between 2012 and 2016, as evidenced by the abrupt rise in MERS-related publications.

Eight publications provided convincing evidence of camel-to-human transmission; sequencing comparisons between the patient and camel samples showed high similarity. The studies employ various methodologies, including virus isolation, sequencing, and epidemiological investigations, to establish a strong relationship between camels and human cases of MERS-CoV infection.

Azhar et al. (17) isolated and sequenced MERS-CoV from a dromedary camel and a patient with laboratory-confirmed MERS-CoV infection. The whole genome sequences of the two isolates were found to be identical. In a second study, the researchers found identical MERS-CoV RNA sequences in an air sample infected with the virus from the camel’s barn (18). Memish et al. (19) analyzed viral sequences from humans and camels, revealing nucleotide polymorphism signatures indicative of cross-species transmission. A farm in Qatar was associated with two human cases of MERS-CoV infection, according to research conducted by Haagmans et al. (16). The authors detected the virus in nasal swabs from three camels. The nucleotide sequences of the camel isolates were highly similar to those obtained from the infected humans on the same farm, indicating a potential outbreak affecting both species. Similarly, Al Hammadi et al. (23) identified asymptomatic MERS-CoV infection in two men exposed to infected dromedaries in the United Arab Emirates. The genetic sequences of MERS-CoV from the men and camels exhibited similarities with those detected in other countries, supporting zoonotic transmission. Al Muhairi et al. (21) conducted a comprehensive study involving 1,113 dromedary camels and two MERS-CoV-infected camel farm owners in the UAE. Sequencing analysis revealed that the camel sequences and sequences from one farm owner clustered within the larger MERS-CoV sequence cluster, further demonstrating the zoonotic odds of MERS-CoV transmission.

Additional studies from Kasem et al. (22), Paden et al. (20), and Ngere et al. (24) provided further evidence of camel-to-human transmission. Kasem et al. (22) found complete genome sequence identity between MERS-CoV isolates. Paden et al. (20) sequenced whole MERS-CoV genomes from respiratory samples collected from human cases and camels in the UAE, finding nearly identical genomes between the two groups. Finally, Ngere et al. (24) demonstrated sporadic transmission of MERS-CoV from camels to humans during intense outbreaks in Kenya. As per the researchers, an analysis of camel swabs collected from calves in Kenya between April and September 2019 yielded interesting results. Of the 4,692 swabs collected from 83 calves in 15 homesteads, 2.6% (124 samples) tested positive for the virus. Additionally, it was observed that 22 calves (26.5%) showed reinfections, indicating a subsequent positive swab after at least two consecutive negative tests. Further investigation through sequencing unveiled the presence of a distinct clade C2 virus. Unlike other clade C viruses, this strain did not exhibit the typical ORF4b deletions. Notably, three previously reported cases of humans testing positive for the virus via PCR clustered temporally and geographically with the camel infections. These findings suggest that sporadic transmission from camels to humans occurred during the peak of camel outbreaks in Northern Kenya. Previous studies have established the widespread occurrence of MERS-CoV in Kenyan camels, confirming its geographical presence (20, 43).

#### 4.1.1. Current gaps and priorities for research

Based on epidemiological and virological studies, the dromedary camel is the most likely source of human MERS-CoV infections. According to some theories, MERS-CoV may have started in bats and then spread to camels (44–46). However, the ancestral origin of MERS-CoV and the exact source and mechanism of direct transmission to humans remain unknown. Comparatively, few publications of MERS-CoV genome analysis from humans and 8 camels have been published (46). Therefore, continuous surveillance and genomic investigations are required to investigate the virus’s spread and evolution among camels and to find the variation of concerns (VOCs) linked with pandemic potential, as in the scenario with SARS-CoV-2. Furthermore, the identification of MERS-CoV in *Hyalomma dromedarii* ticks attached to dromedary camels in the United Arab Emirates (47) has raised intriguing issues about the potential role of arthropod vectors in the disease’s transmission. Consequently, it would be interesting to determine if *H. dromedarii* is a competent vector for MERS-CoV, and its potential significance in mechanical or fomite transmission between camels should be explored. Additionally, research directed towards developing vaccines for camels to limit spillover infections to humans, validation of the current diagnostic techniques, and active surveillance of MERS-CoV in camels would be of great value in implementing sound mitigation interventions.

### 4.2. Brucellosis

The bacterial disease known as brucellosis, which can affect both humans and animals, is caused by many species of the genus *Brucella*. It is considered one of the most common neglected zoonotic diseases, primarily impacting the reproductive systems. In animals, infection with Brucella is characterized by signs such as abortion, infertility, retained placenta, orchitis, epididymitis, and, uncommonly, arthritis. The organisms can be excreted in uterine discharges, milk, urine, and semen (48).

Human brucellosis is mainly transmitted by consuming contaminated raw dairy products and meat from domestic animals. Except for Australia, camel brucellosis cases have been recorded in almost all countries raising camels. Some countries are experiencing an increase in the prevalence of the disease among camels due to unrestricted trade in live animals and inadequate measures to control its spread.

Evidence of disease transmission from camels to humans has been documented in five publications from the United Arab Emirates (UAE), the Horn of Africa, Qatar, and Israel. In a notable case in the UAE, Schulze zur Wiesch et al. (25) reported the diagnosis of acute brucellosis in a veterinarian working in a veterinary laboratory. The genomic analysis identified matching brucella strains in dromedary milk samples and patient cultures, supporting the diagnosis of laboratory-acquired infection. This study emphasized the high brucellosis infectivity, including airborne transmission, in veterinary laboratories handling camel specimens, highlighting the need for robust biosafety measures. Another study by Shimol et al. (26) described an outbreak of human brucellosis in Israel, where drinking camel milk was identified as the mode of infection. In this case, 15 patients were diagnosed with acute brucellosis, and *Brucella melitensis* was confirmed in their blood cultures and the camel’s milk through serology and culture. Whole-genome sequencing in Israel linked patients with *B. melitensis* to uncontrolled livestock trading and wholesale camel milk (27).

Previously, reports implicating camel milk as a source of brucella infection were limited to patients residing in or traveling to and from the Middle East. However, Rhodes et al. (28) reported invasive human brucellosis infections in travelers and immigrants from the Horn of Africa (Somalia, Ethiopia, and Djibouti), highlighting the consumption of raw camel milk as the source of infection. Furthermore, Garcell et al. (29) reported an outbreak of human brucellosis in a rural area in Qatar, where 14 family members who owned camels and sheep were affected. The source of the infection was identified as the milk of an infected camel, which was confirmed through serological studies conducted on the patients.

These studies collectively contribute to the understanding of camel-associated brucellosis and affirm the importance of considering camels as potential sources of zoonotic diseases, mainly through the consumption of camel milk.

#### 4.2.1. Current gaps and priorities for research

According to several researchers, breeding camelids experience fewer abortions due to brucella infection than cattle and small ruminants (49). Abu Damir et al. (50) experimentally infected camels with bacterial strains (S19 and a field bovine strain) in the only publication of its kind. It is, therefore, necessary to experimentally infect camels with camel-isolated *Brucella* strains to understand the pathogenesis, pathology, clinical symptoms, and bacterial shedding.

Future studies should focus on determining the pathology and pathogenesis of *Brucella* strains in experimentally infected camels, assessing the risk of zoonotic infection in laboratories, and evaluating immunization strategies. Abbas and Agab (51) proposed endeavoring vaccination of camels with *B. abortus* strain RB51 in this context. This strain was tried successfully in adult cattle and bison with many benefits, including a lack of interference with serological diagnosis. The authors also suggested bacteriologic surveys to conclude the relative importance of brucella species (*B. melitensis* and *B. abortus*) in the etiology of camel brucellosis.

### 4.3. Camel plague (*Yersinia pestis* infection)

*Y. pestis*, an anaerobic bacterium, is responsible for causing the human plague or the Black Death, a fatal disease spread by flea bites from naturally infected rats to people. However, camels and other mammals can contract the disease (52). During the middle-age, plague pandemics claimed the lives of approximately 200 million people. Although the intensity has significantly decreased, the natural foci of plague persist in many locations around the globe, particularly in Africa and Asia. Camelids (Dromedary and Bactrian camels, New World Camelids) are susceptible to *Y. pestis*, and plague cases have been reported in these animals in different regions where these animals are reared. In contrast to other agricultural animals, the wide-ranging behavior of camels enhances their tendency to come into contact with natural habitats of plague (33). Ancient Arabs believed that the excessive death of camels was a warning of an approaching human plague because, in the past, many humans had contracted the disease from camels (52). From the former USSR, there have been several cases of *Y. pestis* spreading from plague-infected camels to people during the early years of the previous century.

Recently, four studies have indicated a connection between *Y. pestis* infection and contact with dromedary camels. In 1997, Arbaji et al. (33) investigated a plague outbreak in a Jordanian village marked by fever and cervical lymphadenopathy. The affected individuals reported consuming raw or cooked meat from the same camel’s carcass. Bin Saeed et al. (31) examined a cluster of five plague cases in Saudi Arabia, including four individuals with severe pharyngitis and submandibular lymphadenitis in 1994. The four patients had consumed raw camel liver. *Y. pestis* was isolated from the camel’s bone marrow, and fleas and jirds were caught in the camel pen. Leslie et al. (32) reported an outbreak of plague in Afghanistan with a rare gastrointestinal presentation associated with consuming or handling camel meat. Seventeen people died as a result of the outbreak. Molecular and immunological testing of patient clinical samples and camel tissue revealed DNA signatures consistent with *Y. pestis*. Christie et al. (30) described an incident in Libya in 1976 where the meat of a slaughtered sick camel was distributed for human consumption. Several days later, 15 villagers fell severely ill with a febrile illness, and those who had participated in the slaughtering and distribution of the camel all died within 4 days. Using the passive hemagglutination test, samples from the remaining patients showed evidence of plague.

In conclusion, evidence suggests that humans can contract *Y. pestis* from dromedary camels. Cases of plague associated with the consumption of camel meat or contact with infected camels have been reported in various regions. This highlights the importance of implementing appropriate preventive measures and raising awareness about the potential risks associated with camel-related zoonotic diseases.

#### 4.3.1. Current gaps and priorities for research

*Y. pestis* is a major meat-borne zoonotic bacterial pathogen, and its management necessitates action at the point where people, animals, and their environments interact. Until recently, plague in camels was diagnosed only after the animal’s death; such a diagnosis was not established in living camels (52). Therefore, research is needed to develop diagnostic tools for rapidly detecting and confirming *Y. pestis* before and post-mortem at slaughterhouses. Limited trials of the anti-plague vaccines in camels have been conducted, and genetically modified vaccines are also recently developed to protect both humans and animals from the plague (52). Vaccination trials in camels should be investigated to determine the best dose, safety, and efficacy. Furthermore, an assessment of risk factors in human and animal populations and the socioeconomic impacts of the disease are required.

### 4.4. Hepatitis E infection

The hepatitis E virus (HEV) is the primary cause of the emerging zoonotic enteric disease known as hepatitis E, and belongs to the *Orthohepevirus* genus in the family *Hepeviridae*. This family comprises four species: *Orthohepevirus A–D*. HEV is primarily transmitted through the fecal-oral route and is well-known as a zoonotic pathogen (53).

Woo et al. (54) reported a novel genotype of HEV identified in dromedaries, suggesting another potential source of human HEV infection. A molecular epidemiology study in Dubai, United Arab Emirates, detected HEV in fecal samples from three camels. Complete genome sequencing of two strains revealed more than 20% nucleotide difference compared to known HEVs. A previously unknown genotype, ultimately identified as HEV7, a novel *Orthohepevirus A* genotype exclusive to dromedaries, was discovered by comparative genomic and phylogenetic analysis (55, 56).

A new HEV genotype was detected in Bactrian camels in 2016 in Xinjiang, China. Sequence analysis demonstrated that the three Bactrian HEV strains represented a distinct genotype, currently classified as HEV8 (57). Despite successfully identifying genotypes 7 and 8 in dromedaries and Bactrian camels, these viruses’ epidemiology, zoonotic potential, and pathogenicity remained unclear.

Only one study has provided evidence of virus transmission from camels to humans. Lee et al. (37) conducted partial and full-length phylogenetic analyses of HEV sequences from a liver transplant patient in the Middle East and compared them with other *Orthohepevirus A* sequences. According to the findings, people who routinely consume camel milk and meat were infected with camelid HEV. The authors concluded that camelid HEV, specifically genotype 7, might be capable of infecting humans.

In conclusion, hepatitis E is an emerging zoonotic disease. Novel genotypes (HEV7 and HEV8) have been discovered in dromedaries and Bactrian camels. While the zoonotic potential of these viruses is not yet fully understood, evidence suggests the transmission of camelid HEV (genotype 7) to humans through camel meat and milk consumption.

#### 4.4.1. Current gaps and priorities for research

Limited knowledge exists regarding the amount of foodborne transmission and the role played by camels in the zoonotic spread of HEV to people. Research is needed to investigate these camel-associated HEV genotypes’ epidemiology, zoonotic transmission dynamics, and pathogenicity.

### 4.5. Camelpox

Camelpox is a highly infectious skin disease and the most encountered viral infection of Old-World camelids (Dromedary and Bactrian camels), endemic in almost every country where camel husbandry is practiced, except for Australia (58). The camelpox virus (CMLV), a member of the *Orthopoxvirus* (OPXV) genus in the *Chordopoxvirinae* subfamily of the *Poxviridae* family, is the disease-causing agent. Camelpox has significant economic implications due to its high mortality rate, weight loss, reduced milk yield, and general deterioration of the condition. Clinically, two distinct types of camelpox can be recognized: the more severe generalized type, which is more common in young animals, and the less severe localized form, frequently seen in older camels (59).

During the smallpox eradication campaign, camelpox was initially considered a potential non-human reservoir of VARV (variola virus), as under particular laboratory circumstances, the two viruses exhibited no discernible differences (60). Although CMLV only causes mild infection in humans and does not spread from person to person, there is also fear that it could be utilized as a biological weapon due to its strong genetic similarity to the variola virus (61).

The first documented case of human camelpox was reported in Somalia (34). A 40 years-old man who had not been immunized against smallpox and had come into contact with diseased animals had a rash on his arms that progressed through vesicular, pustular, and scab stages. Testing conducted at the Sera and Vaccine Institute in Mogadishu confirmed the presence of *orthopoxvirus* antibodies through a passive hemagglutination inhibition test using serum from the patient. Samples taken from sick animals in the patient’s group tested positive for *orthopoxvirus* by electron microscopy, and the camelpox virus was isolated (34).

In 2009, outbreaks of dromedary camels in northwest India, reported by Bera et al. (35), provided the first strong proof of human zoonotic camelpox virus (CMLV) infection. Three human cases of CMLV zoonosis were confirmed using clinical and epidemiological attributes, serological tests, and molecular characterization of the causal agent. The camel handlers’ hands and fingers were the only sites for the lesions, which developed through all stages of pock lesions until the development of scabs (35). Notably, none of the patients in the three suspected cases had received the smallpox vaccine. Serum samples from these patients revealed neutralizing antibodies against CMLV. In one of the three human cases, viral DNA specific to CMLV was detected using conventional PCR.

By describing cases involving dromedary camels and three camel herders in the Showak region of eastern Sudan between September and December 2014, Khalafalla and Abdelazim (36) offered a second piece of evidence proving the zoonotic nature of the camelpox virus. Erythema, vesicles, and pustules on the arms, hands, legs, back, and abdomen were the main skin lesions in the camel herders; they disappeared after 2 months without being spread from person to person. The diagnosis was verified by PCR, virus isolation in cell culture, and partial genome sequencing. Due to the relatively mild nature of camelpox in humans and the limited ability of the virus to spread among people, the risk to human health from this rare infection is currently considered low.

#### 4.5.1. Current gaps and priorities for research

Events of zoonotic transmission of camelpox were documented between 1978 and 2014 in geographically remote areas of Somalia, India, and Sudan. Therefore, it is crucial to determine the factors that contributed to the emergence of the disease by isolating and sequencing CMLV strains from human patients. Studying the epidemiology of camelpox through active surveillance in regions where zoonotic transmission has occurred, such as the Showak region of eastern Sudan, is the focus of this research. Serological surveys involving dromedary camels, their shepherds, and humans who come into contact with camels in livestock markets and slaughterhouses through a one-health approach could be instrumental in tracking down and investigating zoonotic incidents.

### 4.6. Anthrax

Anthrax is a zoonotic bacterial disease caused by *Bacillus anthracis*, which leads to severe illness and death in humans, livestock, and wild animals. Infected animals often succumb to the disease without showing any signs of disease. Humans can contract anthrax by handling contaminated animal products, consuming undercooked meat from infected animals, and, in recent cases, the intentional release of spores.

A significant report by Musa et al. (38) provided epidemiological evidence of camel-to-human transmission of anthrax. The study investigated an anthrax outbreak in camels that resulted in 10 human infections in Darfur, western Sudan. Five affected individuals died, while the others received successful treatment. The disease was diagnosed based on human symptoms and through the Ascoli’s precipitation test in camels. Control measures were implemented by mass vaccination of animals in the affected area.

In conclusion, timely diagnosis and effective control measures to mitigate the spread of this disease could be of great value. Vaccination programs are crucial in preventing anthrax outbreaks in animals and minimizing the risk of transmission to humans. Public awareness about handling and cooking animal products is essential for reducing the likelihood of human infections.

#### 4.6.1. Current gaps and priorities for research

As demonstrated in the mentioned outbreak, the spread of anthrax from infected camels to humans highlights the importance of disease surveillance and risk assessment to establish a baseline for control measurement.

## 5. Additional zoonotic diseases that camels could transmit

### 5.1. Toxoplasmosis

Toxoplasmosis is a parasitic disease caused by *T. gondii* and is prevalent in various animals, including camels. Tonouhewa et al. (62) highlighted the widespread presence of *T. gondii* in camels, raising concerns about food safety in African countries. While the presence of *T. gondii* tachyzoites in camel’s milk was documented, a direct epidemiological link to confirmed human cases was not established.

Humans can become infected with *T. gondii* through various means, such as consuming undercooked or raw meat, ingesting oocysts shed by cats through contaminated soil, food, or water, or even through transmission from mother to fetus during pregnancy. Previous studies have suggested that milk, including camel’s milk, could be a source of infection. In one particular study conducted in the Butana area of eastern Sudan, researchers investigated the role of camel’s milk in human toxoplasmosis. The study by Medani and Mohamed (63) presented at the 17th International Congress on Infectious Diseases examined the presence of *T. gondii* tachyzoites (a stage in the parasite’s life cycle) in camel’s milk. Ten milk samples from infected camels were utilized to perform an IgM anti-*T. gondii* ELISA to confirm the infection. These milk samples were then inoculated into naive kittens and mice. The results demonstrated that all the inoculated animals shed Toxoplasma oocysts, and ELISA testing confirmed the infection. However, it is essential to note that while this study suggests a possible link between camel’s milk and human toxoplasmosis, it does not provide conclusive evidence. Additionally, the high seroreactivity of Toxoplasma observed among camel herders in the Butana area of eastern Sudan, as reported by Khalil et al. (64), raises concerns about the public health implications for Sudanese nomads who consume raw camel milk.

### 5.2. Rift Valley fever

Rift Valley fever (RVF) is an acute disease transmitted by arthropods and caused by the RVF virus (RVFV), which belongs to the *Bunyaviridae* family and is primarily transmitted by mosquitoes. The disease was initially observed in Kenya in 1930 and has since experienced periodic outbreaks in small ruminants and cattle. These outbreaks have also led to the spread of the disease to humans in sub-Saharan Africa and the Arabian Peninsula (65). During an RVF outbreak in northeastern Kenya in 1962, camels were identified as susceptible to the virus for the first time, expanding the list of affected animal species. In September 2010, a significant RVF outbreak occurred in northern Mauritania, resulting in mass abortions among small ruminants and dromedary camels and at least 63 human clinical cases, including 13 fatalities. Among camels, the serological prevalence of the virus ranged from 27.5% to 38.5%. Notably, this outbreak marked the first clinical signs beyond abortions in camels, with animals exhibiting symptoms such as hemorrhagic septicemia and severe respiratory distress (66). During the 2022 outbreak in Mauritania, there were 47 confirmed cases of RVF, predominantly among animal breeders, with 23 fatalities reported (67). The presence of the RVF virus in animals, including small ruminants, camels, and cattle, was confirmed, with 25.8% of camel samples testing positive through RT-PCR, compared to 5.2% in cattle and 25.9% in small ruminants. This result highlighted the role played by camels in the spread and transmission of RVF, which is roughly equal to the role of small ruminants.

### 5.3. Tuberculosis

In a study by Gumi et al. (68), samples were collected from pastoralists in Ethiopia’s Oromia and Somali Regional States with suspected tuberculosis (TB) and tuberculous lesions collected from cattle, camels, and goats at abattoirs. Culturing of humans yielded several *Mycobacterium tuberculosis* isolates, and molecular typing confirmed these isolates as *Mycobacterium bovis* and non-tuberculous mycobacteria (NTMs). Similarly, several isolates were obtained from tuberculous lesions of livestock, of which one *M. tuberculosis* and one NTM from camels. The authors concluded that the isolation of *M. tuberculosis* from livestock and *M. bovis* from humans indicates transmission between livestock and people in South-East Ethiopia’s pastoral districts.

### 5.4. Crimean-Congo hemorrhagic fever

Crimean-Congo hemorrhagic fever (CCHF) is a tick-borne disease caused by the CCHF virus (CCHFV) that causes moderate to severe hemorrhagic disease in humans with high case fatality ratios of up to 40%. The virus spreads more quickly when infected ticks move to new, uninfected areas since the distribution of CCHFV correlates with that of its primary vector, ticks of the genus *Hyalomma*. Recently, a new lineage of CCHFV with potential genome reassortment of the M segment was found in dromedary camels and camel ticks (*Hyalomma dromedarii*) (69, 70).

### 5.5. Q fever (query fever, Coxiellosis)

Q fever, a significant zoonotic disease, is caused by the bacterium *Coxiella burnetii*. Infection can occur when individuals inhale aerosolized organisms or through other routes. Human infections can range from asymptomatic to causing acute, nonspecific febrile illness, often accompanied by hepatitis and atypical pneumonia (71). While there has been extensive research on the zoonotic impact of *C. burnetii* infection in small ruminant and cattle populations, the potential role of camels in transmitting Q fever to humans has received limited attention until recently (72). Given the favorable conditions for the pathogen, the Middle East region faces a significant public health threat from Q fever. Camels have an overall seroprevalence of 25% for *C. burnetii* (73). However, a study by Hussein et al. (74) in Saudi Arabia using ELISA and indirect immunofluorescence (IFA) tests found antibodies to *C. burnetii* in 51.64% of camel serum, indicating a substantially higher proportion. The authors also conducted PCR analysis on clinical samples from seropositive camels, detecting positive DNA amplification. The highest shedding of *C. burnetii* was found in fecal samples (27.59%), followed by urine (23.81%), blood (15.85%), and milk (6.5%). Based on these findings, the authors concluded that camels are a significant reservoir for *C. burnetii* and can be a primary source of Q fever transmission to humans in Saudi Arabia. In Africa, higher seropositivity rates for Q fever in camels have been reported, with 38.6%, 73.6%, and 75.5% in Kenya, Tunisia, and Algeria, respectively (75–77).

### 5.6. Trypanosomiasis

*Trypanosoma evansi* is a blood parasite found in South America, North Africa, the Middle East, and South and Southeast Asia that causes acute disease in camels and horses (surra) and chronic disease in cattle and buffalo (78). Although *T. evansi* cases in humans have been documented in India and Egypt (79), there is no epidemiological or molecular evidence that these human cases are related to the camels, in whom this parasite is known to cause a devastating and economically significant disease.

### 5.7. Camel contagious ecthyma

Contagious ecthyma (CE), also known as orf, represents an acute, highly infectious disease caused by different virus species of the genus *Parapoxvirus* (PPV) and family *Poxviridae.* Parapoxviruses (PPVs) commonly cause infectious skin diseases primarily affecting ruminants and other animal species, including the dromedary camel. These viruses cause proliferative exanthematous dermatitis, typified by the formation of pustules and scabs predominantly localized on the oral mucosa of afflicted animals.

It is noteworthy that the disease exhibits a more severe clinical course in camels compared to its manifestations in sheep or goats, often culminating in a heightened case fatality rate (80). Most PPVs are thought to be zoonotic (81). Sheep-to-humans and goats-to-humans transmissions of ORFV have been documented in the literature (82, 83). Conversely, the transmission of the infection from camels to humans has not been previously reported, a position supported by empirical field investigations suggesting that CE in camels is not of zoonotic concern (80). Nevertheless, our literature examination unveiled an article detailing an intriguing case, suggesting potential camel-to-human transmission of CE (84). According to the authors, a 42 years-old male who had direct contact with a sick camel exhibited clinical manifestations consistent with orf characterized by multiple erythematous, dome-shaped to round painless nodules on the right forearm, further complicated by the occurrence of lymphadenopathy. The diagnostic determination of orf was rendered primarily based on clinical suspicion. It is noteworthy that the patient in question routinely engaged in the care of his camels and occasionally milked them. Significantly, one of his camels bore signs of CE in the form of a rash surrounding its oral cavity and both lips.

Based on the available data, classifying this case as a zoonotic camel-to-human transmission is challenging because there is no test confirmation of the suspected CE lesion on the patient and his camel. However, the publication highlighted the importance of CE and pointed out that the transmission of CE infection from camels to humans needs to be studied. Such events may go unreported because the infection is self-limiting and because those affected are frequently aware of their condition and do not seek treatment.

## 6. Comments on the additional zoonotic diseases that camels could transmit

In conclusion, camels have been associated with several other zoonotic diseases, including toxoplasmosis, Rift Valley fever, TB, Crimean-Congo hemorrhagic fever, Q fever, trypanosomiasis, and camel contagious ecthyma despite having no evidence of a laboratory-confirmed transmission event. These findings emphasize the need for comprehensive surveillance, preventive measures, and public health interventions based on a One Health approach to mitigate the risks of zoonotic infections linked to camels. Proper handling and processing of camel-derived products and implementing vaccination programs and vector control strategies are essential for reducing the transmission of these diseases to humans.

## 7. Mitigating risks and ensuring public health

To address the risks posed by camel-associated zoonotic diseases, it is necessary to increase understanding and awareness among medical professionals, veterinary authorities, and the general public. Camel herds should be under surveillance for zoonotic infections, and preventative measures like hygienic practices and appropriate food safety rules should be implemented. Collaboration between the human and animal health sectors is crucial to prevent and control zoonotic diseases.

## Author contributions

The author is solely responsible for the conceptualization, design, data collection, analysis, and interpretation of the study, as well as the writing and formatting of the manuscript.
